# Dengue Virus Neutralization in Cells Expressing Fc Gamma Receptors

**DOI:** 10.1371/journal.pone.0065231

**Published:** 2013-05-22

**Authors:** Tanu Chawla, Kuan Rong Chan, Summer L. Zhang, Hwee Cheng Tan, Angeline P. C. Lim, Brendon J. Hanson, Eng Eong Ooi

**Affiliations:** 1 Program in Emerging Infectious Diseases, Duke-National University of Singapore Graduate Medical School, Singapore, Singapore; 2 Defence Science Organization National Laboratories, Singapore, Singapore; University of Rochester, United States of America

## Abstract

Activating Fc gamma receptors (FcγRs) in hematopoietic cells serve to remove antibody-opsonized antigens, including dengue virus (DENV), from systemic circulation. While neutralizing antibody concentrations provide humoral immunity, cross-reactive or sub-neutralizing levels of antibody can result in antibody-dependent enhancement of DENV infection that increases overall viral burden. Recently, it has been suggested that the antibody levels needed for DENV neutralization differs when different FcγR is engaged. If this is true, the threshold titer used to infer immunity should be influenced by FcγR usage. Here, using cells that express both activating and inhibitory FcγRs, we show that the type of FcγR engaged during phagocytosis can influence the antibody concentration requirement for DENV neutralization. We demonstrate that phagocytosis through FcγRI requires significantly less antibody for complete DENV neutralization compared to FcγRIIA. Furthermore, when DENV is opsonized with sub-neutralizing levels of antibody, FcγRI-mediated phagocytosis resulted in significantly reduced DENV titers compared to FcγRIIA. However, while FcγRI may remove antibody-opsonized DENV more efficiently, this receptor is only preferentially engaged by clustering when neutralizing, but not sub-neutralizing antibody concentrations, were used. Collectively, our study demonstrates that activating FcγR usage may influence antibody titers needed for DENV neutralization.

## Introduction

Dengue is the most common mosquito-borne viral disease globally. It is caused by a positive-strand RNA virus, which exists as four antigenically distinct serotypes. Infection with dengue virus (DENV) results in a spectrum of illness that ranges from undifferentiated fever to severe dengue that comprises hypovolemic shock from plasma leakage, internal hemorrhage or organ dysfunction. While antibody response triggered during the acute infection result in lifelong immunity to the homologous serotype, infection with a heterologous DENV serotype or during a time where maternally acquired antibodies wane in infants have been shown to be epidemiologically associated with increased risk of severe dengue [1]–[3]. Cross-reactive or sub-neutralizing levels of antibodies offer DENV with an alternative pathway of entry into monocytes, macrophages and dendritic cells through the activating Fc gamma receptors (FcγRs). This pathway of infection, termed antibody-dependent enhancement of DENV infection (ADE), is hypothesized to be an important mechanism in the pathogenesis of severe dengue [3]–[7]. FcγRs are broadly expressed by cells of hematopoietic origin and is composed of activating (FcγRI, FcγRIIA, and FcγRIIIA) and inhibitory (FcγRIIB) receptors [8]. While these receptors could contribute to ADE [9], [10], they are important in the removal of DENV opsonized with neutralizing levels of antibody. Delineating the determinants of neutralization or ADE upon FcγR-mediated phagocytosis would thus be important for the understanding of immunity and pathogenesis, respectively, which could prove useful in refining vaccine development to overcome the currently observed limited immunity with the leading dengue vaccine candidate [11].

Stoichiometric studies have shown that neutralization of flavivirus is a “multi-hit phenomenon”, which occurs when the number of antibodies bound to a virus exceeds a required threshold and is dependent on antibody affinity and epitope accessibility [12]–[14]. However, the stoichiometric requirement for DENV neutralization may be different when phagocytosis is mediated by either FcγRI or FcγRIIA. Rodrigo and colleagues used a panel of monoclonal antibodies to demonstrate that DENV neutralization required significantly lower antibody concentration in CV-1 cells transfected with FcγRI compared to FcγRIIA [15]. However, the gamma subunit containing the immunoreceptor tyrosine activating motif that signals for phagocytosis upon was covalently linked FcγRI in the transfected cells whereas in cells that naturally express this receptor, the gamma subunit is only recruited upon activation of the receptor [16]. Whether the experimental design adopted by Rodrigo and colleagues [15] affected the outcome of the antibody concentration needed for complete DENV neutralization, is unknown. We hence utilized cells that naturally express FcγRs to investigate the antibody concentration requirements for DENV neutralization. We show here that more antibodies are required for DENV neutralization with FcγRIIA- compared to FcγRI-mediated phagocytosis. Furthermore, when both receptors are expressed together, DENV opsonized with neutralizing levels of antibody preferentially engage FcγRI by clustering this receptor on the cell membrane.

## Materials and Methods

### Cells and Antibodies

BHK-21, THP-1, K562 and Vero cells were purchased from the American Type Culture Collection (ATCC) and cultured according to ATCC recommendation. 3H5 is a monoclonal antibody that binds to domain III of DENV envelope protein. A chimeric human antibody of 3H5 (h3H5) IgG1 was constructed consisting of mouse VH and VL sequences and human γ1 and κ constant sequences [17]. These antibodies were indistinguishable from the parent 3H5 mAb in their ability to bind to DENV-2 [18]. Antibodies used for flow cytometry staining, western blot and immunofluorescence assay (IFA) were: FcγRI antibody clone 10.1 (eBioscience), FcγRII clone IV.3 (Stem cell biology), FcγRIIB (Abcam), LAMP-1 (BD biosciences, Abcam), Cy3 anti-LAMP-1 (Sigma) and HRP conjugated anti-mouse (Dako). All Alexa Fluor labeled antibodies were purchased from Invitrogen and used at 1∶200 dilution.

### Virus culture and purification

DENV-2 (ST strain) was first isolated from a clinical sample from Singapore General Hospital. Viruses were propagated in Vero cell line and harvested 5 days post infection (dpi) and purified through 30% sucrose. Virus pellets were resuspended in 5 mM Hepes, 150 mM NaCl, and 0.1 mM EDTA (HNE) buffer, aliquoted and stored at −80°C until use. Infectious titer was determined by plaque assay.

### Plaque Assay

Ten fold serial dilutions of virus culture supernatant were added to monolayer of BHK-21 cells in 24-well plates and incubated for 1 h at 37 °C with gentle rocking every 15 mins. The inoculum was aspirated, replaced with 0.8% methyl-cellulose in maintenance medium (RPMI-1640, 2% FCS, 25 mM Hepes, penicillin, and streptomycin) and incubated at 37 °C. After 5 dpi, cells were fixed with 20% formaldehyde at room temperature for 20 mins, washed with water, and stained with 1% crystal violet for additional 20 mins. The plates were washed, dried, and the plaque forming units per milliliter (pfu/mL) were calculated.

### Titration of h3H5 antibody for complete neutralization in THP-1 or K562 cells

Two fold serial dilutions of h3H5 (200 µg/mL to 0.097 µg/mL) were incubated with DENV at a multiplicity of infection (MOI) 10 for 1 h at 37°C and then added to THP-1 or K562 cells, subjected to synchronization for 20 mins on ice and then incubated at 37°C for 72 h. The virus culture supernatants were harvested and quantified by plaque assay. The antibody dilution required to mediate full virus neutralization was then determined using the following formula:% neutralization  =  {[virus only (pfu) – (virus + antibody) (pfu)]/virus only (pfu)} ×100

### Fluorescent Labeling of Viruses

DiD (1, 1′-dioctadecyl-3, 3, 3′, 3′-tetramethylindodicarbocyanine, 4-chlorobenzenesulfonate salt) labeling of DENV was done as previously described [18]-[20].Briefly, ∼2×10^8^ pfu purified DENV was mixed with 800 nmol of DiD (Invitrogen) in DMSO (final DMSO concentration <2.5%). After 30 mins, free DiD was removed by gel filtration on a Sephadex G-25 column (Amersham Biosciences) equilibrated in HNE buffer. DiD-labeled DENV (DiD-DENV) was stored at 4°C and used within 2–3 h.

DENV was labeled with Alexa Fluor as described previously [21]. Briefly, ∼9×10^8^ pfu purified DENV was incubated with 100 µM of Alexa Fluor 488 (AF488) succinimidyl ester (Invitrogen) for 1 h at room temperature. The labeling reaction was then stopped by adding 1.5 M hydroxylamine, pH 8.5, and incubated at room temperature for 1 h. The excess dye was then removed by gel filtration on a Sephadex G-25 column. AF488-labeled DENV (AF488-DENV) was stored in 100 µL aliquots at −80°C, re-titrated by plaque assay, and tested for fluorescence using IFA on Vero cells before using in experiments.

### Infection for localization studies in THP-1 or K562 cells

Concentrations of h3H5 required for complete neutralization in THP-1 (3.125 µg/mL) or K562 (25 µg/mL) were incubated with DiD-DENV (MOI 10) for 1 h at 37°C. The immune complexes were added to cells, synchronized on ice for 20 mins and incubated for 30 mins at 37°C. Cells were then fixed with 3% paraformaldehyde (PFA) in 1× PBS for 30 mins at 4°C. Fixed cells were processed for IFA.

Neutralizing (3.125 µg/mL) or sub-neutralizing (0.390 µg/mL) concentrations of h3H5 were incubated with AF488-DENV (MOI 10) for 1 h at 37°C. Infection was then carried out in THP-1 for 15, 30, 60 and 120 mins as mentioned above. The infected cells were then fixed and sorted using Fluorescence-activated cell sorting (FACS) before processing for IFA.

### Immunofluorescence Assay (IFA)

Fixed cells were spun onto positively charged microscope slides using a cytospin. Cells were dried, washed with 1× PBS and permeabilized with permeabilizing buffer (0.1% Saponin, 5% BSA in 1XPBS). For experiments using DiD-DENV, permeabilized cells were incubated with mouse anti-human LAMP-1 (1∶500) and stained with AF488 anti-mouse and AF555 anti-human IgG antibodies. For experiments using AF488-DENV, permeabilized cells were incubated with mouse anti-human FcγRI (1∶100) or FcγRII (1∶300) and stained with AF633 anti-mouse IgG and Cy3 anti-LAMP-1 (1∶100) antibodies. Subsequently, cells were washed in 1XPBS, rinsed once with deionized water, dried and mounted with Mowiol 4-88 (Calbiochem, San Diego, CA) with 2.5% Dabco (Sigma–Aldrich, Singapore). Processed cells were then visualized using LSM710 Carl Zeiss Confocal microscope at 63× magnification. 8 representative fields were conveniently selected using confocal microscopy to determine the mean percentage of DiD-DENV positive cells at complete neutralizing conditions.

To quantify co-localization of AF488-DENV with FcγRs, 10 cells were selected to calculate percentage co-localization of DENV with FcγRI or FcγRII at 120 mins post infection by overlap coefficient using Zen 2009 software. The mean intensity of FcγRI or FcγRII when co-localized with DENV was evaluated using a tool Histo in Zen 2009 software for 15, 30, 60 and 120 mins post infection. An area of 70.5±0.28 µm^2^ was analyzed on each cell selected from 10 different fields for all time points.

### Fluorescence-activated cell sorting (FACS)

THP-1 infected with AF488-DENV reacted with neutralizing, sub-neutralizing antibody or in absence of antibody for different time points (15, 30, 60 and 120 mins) of infection was fixed and sorted using BD FACS Aria II cell sorter at Duke-NUS FACS facility. The AF488 positive sorted cells were then subjected to IFA.

### siRNA transfection in THP1 or K562

siRNA knockdown studies in THP-1 have been previously described [18]. 50 nM of human FcγRI or FcγRIIA siRNA (Qiagen) or All-Stars scrambled control siRNA (Qiagen) were used for the knockdown studies. For K562, studies were performed with slight modifications. Human FcγRIIB siRNA (Qiagen) or All-Stars scrambled control siRNA (Qiagen) (50 nM) were incubated with DharmaFect2 (Dharmacon) in serum-free media for 20 mins and then added to cells at a density of 2×10^5^ cells/mL. After 6 h incubation, cells were replaced with RPMI supplemented with 10% fetal calf serum (FCS) for 24 h to allow recovery. This was followed by a second round of siRNA transfection. Knockdown efficiency was determined by western blot or flow cytometry.

### Flow Cytometry to determine surface expression of FcγRs

THP-1 or K562 cells were stained with FcγRI or FcγRII antibody for 30 mins on ice, washed three times using 1XPBS with 1% FCS followed by 30 mins of staining with secondary antibody, AF488 anti-mouse IgG, on ice. After final washes using 1XPBS supplemented with 1% FCS, FACS data acquisition was performed on a BD LSR Fortessa.

### Western Blot

Cells were washed once in 1XPBS and lysed in 1% NP-40 with protease inhibitor (Sigma). The cell lysates were centrifuged to remove insoluble aggregates, mixed with loading buffer and separated by SDS-PAGE before transferring to PVDF (Millipore). FcγRIIB and LAMP-1 were detected with specific antibodies followed by addition of anti-mouse IgG–horseradish peroxidase (HRP). Bands were visualized using ECL (Amersham) for chemiluminescence development.

### Statistical Analysis

Two-tailed unpaired Student's t-test or one-way ANOVA were done using GraphPad Prism v5.0. Results with P<0.05 were considered significant.

## Results

### FcγRIIA-mediated phagocytosis requires increased antibody concentration for DENV neutralization

**Figure 1:**
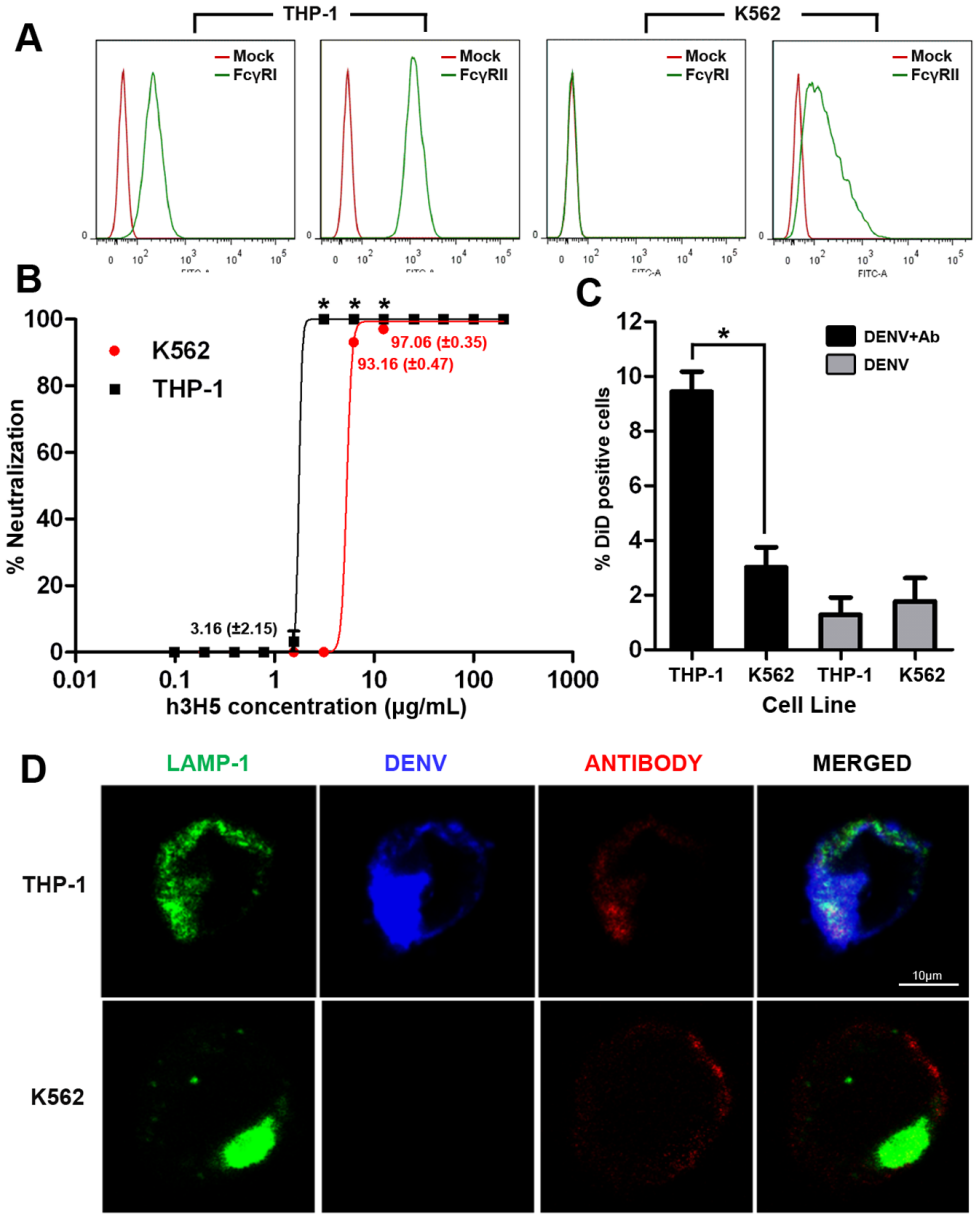
Absence of FcγRI engagement is associated with increased antibody requirement for DENV neutralization. (A) Flow cytometry data of both FcγRI and FcγRII (green histogram) in THP-1 and K562. Mock (red histogram) represents staining with secondary antibody only. (B) Neutralization profile of DENV using various concentrations of h3H5 antibody in THP-1 (Black) and K562 (Red) at 72 h post infection, quantified by plaque assay. Unless indicated, the mean value of neutralization is either 0% or 100%. (C) Percentage of internalized DiD-DENV (% DiD positive cells) in complex with h3H5 antibody is represented by black bar at concentrations that mediated complete neutralization in THP-1 (3.125 µg/mL) and K562 (25 µg/mL), DiD-DENV without antibody is represented in grey bar, as assessed by confocal microscopy at 30 mins post infection. (D) Subcellular localization of DiD-DENV opsonized at h3H5 antibody concentrations required for complete neutralization in THP-1(3.125 µg/mL) and K562 (25 µg/mL). LAMP-1 is in green, DiD-DENV is in blue and h3H5 antibody is in red. Scale bar is 10 µm. Data are represented as mean ± s.e.m. * p<0.01. Results presented are mean of three independent experiments, each with biological triplicates.

We recently reported the use of humanized 3H5 monoclonal antibody (h3H5) to investigate FcγR-mediated phagocytosis in THP-1, a human monocytic cell line that expresses both FcγRI and FcγRIIA [18]. However, titration of h3H5 in K562, a human myelogenous erythroleukemic cell line that expresses FcγRIIA but not FcγRI (Figure 1A) required eight-fold more antibody for complete DENV neutralization compared to THP-1 (Figure 1B). Using DiD labeled DENV that emits fluorescence only upon phagocytosis [19], [20], we observed that neutralization of DENV in K562 occurred at an antibody concentration where FcγR-mediated phagocytosis was inhibited as indicated by reduced DiD positive cells (Figure 1C and 1D). These findings suggest the h3H5 concentration required for complete DENV neutralization in K562 coincides with that which aggregates DENV to co-ligate FcγRIIB that inhibits phagocytosis, a mechanism that we demonstrated recently [18].

**Figure 2:**
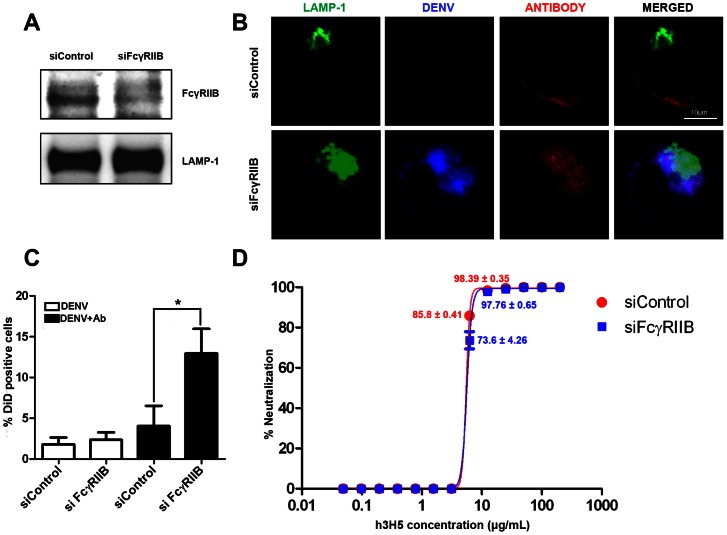
FcγRIIB knockdown did not result in additional increase in antibody requirement for DENV neutralization. (A) K562 transfected with either control siRNA or FcγRIIB siRNA. The reduction in FcγRIIB expression was detected in cell lysate by western blot. LAMP-1 served as a loading control. (B) Subcellular localization of neutralized DENV immune complexes in K562 treated with either control siRNA or FcγRIIB siRNA at 30 mins post infection. LAMP-1 is in green, DiD-DENV is in blue and h3H5 antibody is in red. Scale bar is 10 µm. (C) Percentage of DiD positive cells in K562 treated with either control siRNA or FcγRIIB siRNA with DiD-DENV using 25 µg/mL antibody (black bar) or without antibody (white bar) after 30 mins post infection, assessed by confocal microscopy (D) Neutralization profile of h3H5 against DENV in K562 treated with either siRNA control (red) or siRNA against FcγRIIB (blue) at 72 h post infection, assessed by plaque assay. Unless stated, the mean percent neutralization is either 0% or 100%. Data are represented as mean ± s.e.m. * p<0.01. Results presented are mean of three independent experiments, each with biological triplicates.

That complete DENV neutralization in K562 coincided with FcγRIIB-mediated inhibition of phagocytosis raises the possibility that an even greater amount of antibody is needed to neutralize DENV if phagocytized by FcγRIIA. To test this possibility, we knocked down the expression of FcγRIIB in K562 using siRNA (Figure 2A). This resulted in increased uptake DiD-DENV opsonized with h3H5 (Figure 2B and 2C). However, plaque assay on the culture supernatant indicated that reduced FcγRIIB expression did not result in a further increase in h3H5 antibody concentration needed for complete DENV neutralization (Figure 2D).

**Figure 3:**
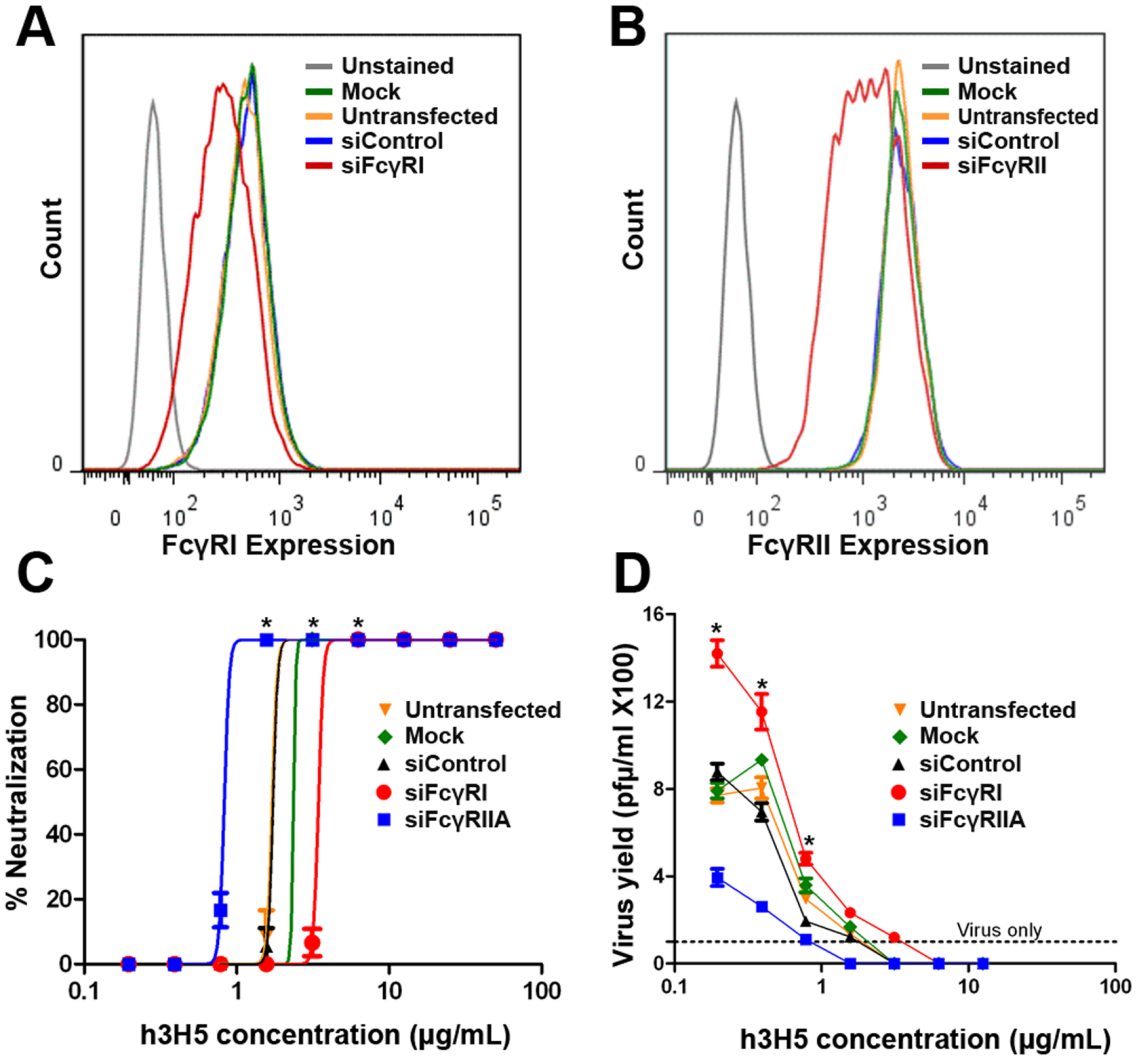
Reduced antibody requirement for neutralization and lowered ADE with FcγRI engagement. (A) Histogram showing surface expression of FcγRI in THP-1 cells when treated with mock transfection (green), control siRNA (blue), FcγRI siRNA (red) or untransfected (yellow). Unstained (grey) indicates negative control stained with secondary antibody only. (B) Histogram showing surface expression of FcγRII in THP-1 cells when treated with mock transfection (green), control siRNA (blue), FcγRI siRNA (red) or untransfected (yellow). Unstained (grey) indicates negative control stained with secondary antibody only. (C) Neutralization profile of untransfected THP-1 cells (yellow) with mock transfection (green), siRNA control (black), siRNA FcγRI (red) or siRNA FcγRIIA (blue), 72 h post infection, as assessed by plaque assay. (D) Virus yield from THP-1 treated with control siRNA, FcγRI siRNA and FcγRIIA siRNA at 72 h post infection, as assessed by plaque assay. Although knockdown efficiency may vary between experiments, we observed similar trends. * p<0.01. Graphs shown are mean ± s.d of biological triplicates of a single experiment from three independent experiments.

As THP-1 and K562 are two different cell lines, we examined if significantly different antibody concentration is needed for DENV neutralization if FcγRI or FcγRIIA expression were respectively altered in THP-1. Reduced expression of FcγRI (Figure 3A and B) resulted in a four-fold increase in the h3H5 concentration needed for DENV neutralization compared to cells with reduced FcγRIIA expression (Figure 3C). Interestingly, reduced expression of FcγRIIA but not FcγRI also resulted in lowered DENV titers even with enhancing levels of h3H5 (Figure 3D). These findings collectively indicate that removal of antibody-opsonized DENV is more efficient with FcγRI than FcγRIIA.

### DENV opsonized with neutralizing but not sub-neutralizing levels of antibody preferentially engage FcγRI

**Figure 4:**
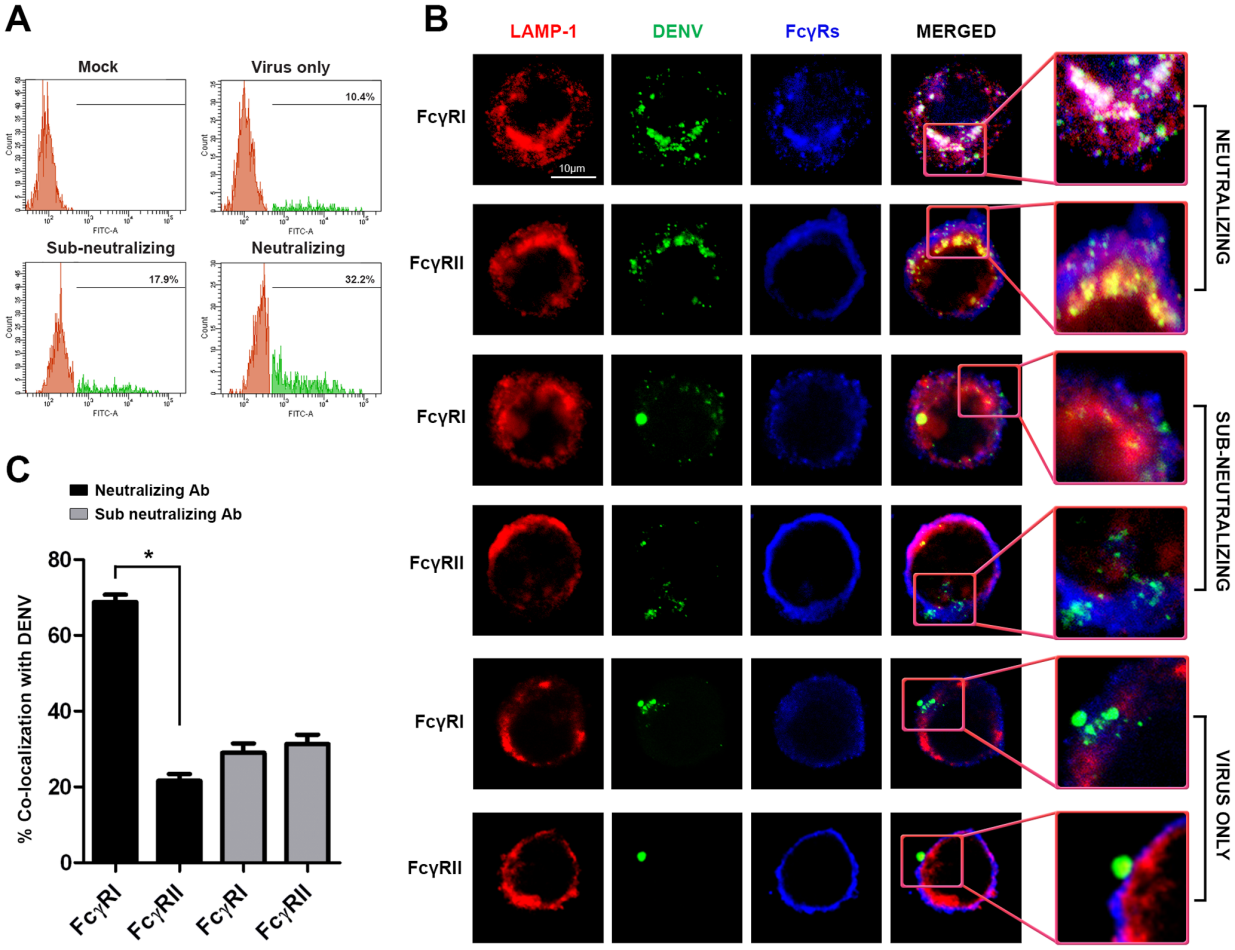
Preferential engagement of FcγRI results in uptake of neutralized DENV immune complexes. (A) Sorting of AF488-DENV infected cells using fluorescence activated cell sorter at 120 mins post infection after synchronization, in absence of antibody (virus only), with neutralizing (3.125 µg/mL) or sub-neutralizing (0.39 µg/mL) antibody concentrations. For mock infection, cells were exposed to h3H5 antibody only. Percentages of AF488-DENV positive cells (green histogram) are numerically indicated. (B) Cellular localization of AF488-DENV immune complexes at neutralizing or sub-neutralizing concentration of h3H5 after 120 mins post infection. LAMP-1 is in red. DENV is in green and FcγRI/FcγRII is in blue. White areas in the merged image indicate the presence of co-localization. Scale bar is 10 µm. (C) Percent co-localization of AF488-DENV opsonized with either neutralizing or sub-neutralizing levels of h3H5, with respect to FcγRI or FcγRII at 120 mins post infection using the confocal microscope, Zen 2009 Software. Images shown are representative of at least 2 separate experiments. Data are represented as mean ± s.e.m. * p<0.01.

Besides reduced antibody concentration requirement, immunofluorescence examination of THP-1 suggests that FcγRI is preferentially engaged by DENV opsonized with neutralizing levels of antibody. As only a subset of THP-1 actively phagocytize antibody-opsonized DENV [18], we enriched for DENV containing cells by sorting for AF488-DENV [21] before affixing the cells on a glass slide for microscopic examination (Figure 4A). At 120 mins post-synchronization, co-localization of DENV, FcγRI and LAMP-1 was observed (Figure 4B). Quantification of the co-localization signals between DENV and FcγRI or FcγRIIA in 10 cells obtained from 10 fields at 63× magnification, using Zen 2009 software indicated a significantly higher co-localization signal with FcγRI than FcγRIIA, but only when neutralizing levels of h3H5 was used (Figure 4C). At sub-neutralizing concentrations of h3H5, however, no difference was observed between the co-localization of AF488-DENV with either FcγRI or FcγRIIA (Figure 4B and 4C). As expected, no co-localization could be observed between either FcγRI or FcγRIIA with DENV only infection.

**Figure 5:**
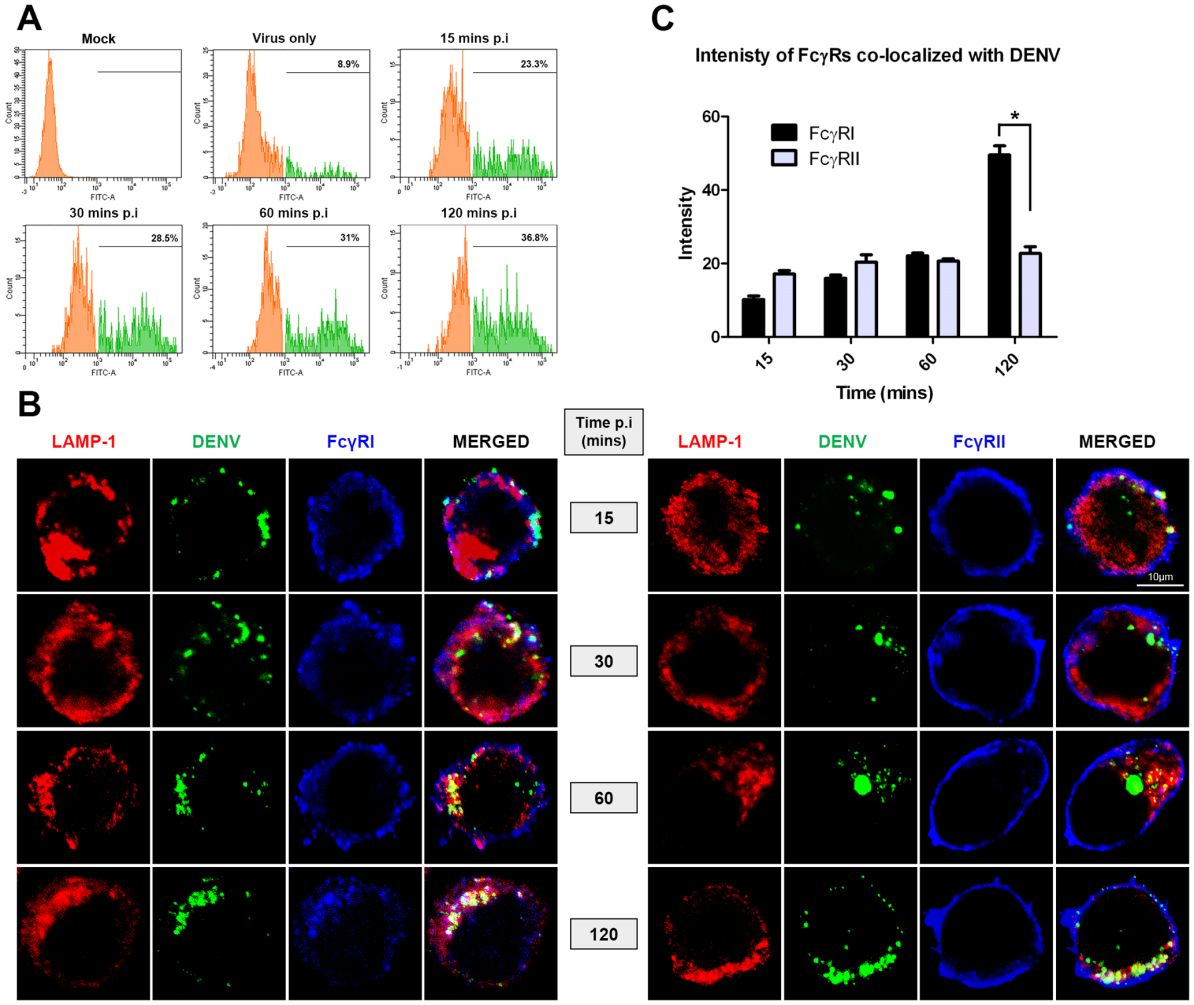
Clustering of FcγRI with neutralized DENV immune complexes. (A) THP-1 infected with AF488-DENV opsonized with neutralizing h3H5 antibody (3.125 µg/mL) were sorted using fluorescence activated cell sorter after 15, 30, 60 and 120 mins post infection (p.i). For mock infection, cells were exposed to h3H5 antibody only. Percentages of AF488-DENV positive cells (green histogram) for different time points are numerically indicated. (B) Cellular localization of AF488-DENV immune complexes, with FcγRI or FcγRII at various time points post infection. LAMP-1 is in red, DENV is in green and FcγRI or FcγRII is in blue. Scale bar is 10 µm. (C) Intensity of FcγRI or FcγRII when co-localized with DENV obtained using the Zen 2009 Software, keeping the selected area (70.5 ± 0.28) µm^2^ consistent for all samples and fields. Statistical test using ANOVA shows a significant increase in intensity of FcγRI with increasing time (p<0.0001) as compared to FcγRII. Images shown are representative of 2 separate experiments. Data are represented as mean ± s.e.m. * p<0.01.

The increased co-localization between DENV and FcγRI suggests that a more efficient pathway is preferentially activated for removal of virus opsonized with neutralizing antibodies. However, the lack of difference in FcγR engagement when sub-neutralizing h3H5 was used is intriguing. Although FcγRI is known to have a greater affinity for IgG1 than FcγRIIA [22], it cannot explain this difference in FcγRI engagement between neutralizing and sub-neutralizing h3H5 since both experiments made use of the same IgG isotype. Instead, the observation may be explained by an antibody-concentration dependent clustering of FcγRI, which has previously been shown as a mechanism to activate this receptor [23], [24]. A time course examination coupled with sorting for cells containing AF488-DENV (Figure 5A) showed increased clustering of FcγRI but not FcγRIIA with increasing time post-synchronization (Figure 5B). To quantify the clustering of FcγRs when co-localized with AF488-DENV, we selected an area of 70.5±0.28 µm^2^ on 10 cells from 10 separate fields under 63× magnification and measured signal intensity of FcγRI and FcγRIIA using Histo tool in Zen 2009 software. Increased FcγRI signal intensity could be observed with increasing time post-synchronization and this was significantly higher than that for FcγRIIA at 120 mins (Figure 5C). This indicates that neutralizing levels of h3H5 was able to cluster and preferentially engage FcγRI for phagocytosis.

## Discussion

Whether antibodies neutralize or enhance DENV infection is determined by both antibody affinity and epitope occupancy [12]–[14]. However most of these studies for DENV neutralization have made use of cells derived from kidney of various animals such as LLC-MK2, Vero, and BHK-1 cells [25]. These cells neither express FcγRs nor are primary targets of DENV in human infections. Recently, it is becoming evident that neutralizing antibody measurements on epithelial cells result in different titers compared to assays that use FcγR-expressing cells [10], [15], [26], [27]. Furthermore, we have also shown that besides blocking specific epitope receptor interaction, antibodies can also aggregate DENV in a concentration-dependent manner to co-ligate the lowly expressed FcγRIIB that inhibits phagocytosis and hence ADE [18]. This also appears to be the mechanism in which neutralization of heterologous DENV serotype occurs [28]. Understanding DENV neutralization in cells that express FcγR thus represents an area for urgent investigation given the recently observed lack of efficacy in vaccines that have relied on traditional virus neutralization test as surrogate of protection [11].

Our findings using cells that naturally express FcγRs corroborate earlier observations that used epithelial cells transfected with FcγRs [15]. We observed that the antibody requirement for DENV neutralization was increased when either K562 or THP-1 with reduced FcγRI expression, was used. In contrast, THP-1 with reduced FcγRIIA expression resulted in reduced antibody requirement for DENV neutralization. We have chosen FcγRI and FcγRIIA for our investigation as they have been previously shown to mediate specific DENV immune complex infectivity in monocytes [6], [7], [10], [29], [30]. FcγRIIIA, on the other hand, is expressed at low levels in a small subset of monocytes [31] and does not affect susceptibility to DENV infection [7]. We demonstrate that depending on whether FcγRI or FcγRIIA mediates phagocytosis, the required threshold of epitopes that must be bound by antibody is different.

FcγRI is an activating receptor that recruits the gamma subunit with immunoreceptor tyrosine-based activating motif to phosphorylate kinases that signal for phagocytosis [16], pro-inflammatory responses [32], protection from bacteria [33] and viruses [34]. Our study highlights the involvement of FcγRI in phagocytosis and neutralization of DENV. Even when DENV was opsonized with enhancing levels of h3H5, phagocytosis through FcγRI produced significantly lower DENV titers. This is consistent with previous report showing that DENV titers were enhanced to a greater effect with FcγRIIA instead of FcγRIA/γ-expressing COS cells [10]. The advantage offered by FcγRI can perhaps be explained by differences in the signaling pathway. A recent study has shown differences in intracellular signaling pathways, receptor trafficking and antigen processing at the early stages between FcγRI and FcγRIIA activation [35]. While both FcγRI and FcγRIIA phagocytize and traffic antibody-opsonized antigens to early endosome compartment (EEA-1), only antigens taken up by FcγRI were trafficked to late endosomal/lysosomal compartments (LAMP-1) [35]. Hence, FcγRI signaling pathways may traffic DENV opsonized with neutralizing levels of antibody into compartments that leads to virus degradation. Conversely, FcγRIIA trafficking may direct DENV into an intracellular environment favorable for replication. Further studies will be needed to substantiate this notion.

That FcγRI possibly offers a more efficient pathway for the clearance of antibody-opsonized DENV also led us to ask how this receptor could be preferentially engaged. We have observed that increased co-localization of DENV with FcγRI relative to FcγRIIA when neutralizing but not sub-neutralizing level of antibody was used. This observation suggests that neutralizing levels of antibody bound on viral surface not only serves to meet the threshold of epitope occupancy, it also clusters FcγRI for preferential activation of this receptor for phagocytosis. It may also be possible that a positive feedback loop exists to augment FcγRI-mediated phagocytosis as activation of FcγRI can induce potent inflammatory response [35] that could increase the clustering of FcγRI and thus binding of immune complexes with it for phagocytosis [23], [24].

Given the role of FcγRI in the clearance of antibody-opsonized DENV suggests that, it is possible that the variable expression of this receptor between different ethnic groups [36] and age [37] could influence the outcome of antibody-enhanced DENV infection. Furthermore, FcγRI expression has been found to be correlated with interferon-gamma (IFNγ) levels [38], which may partially explain the observed reduced ADE in IFNγ-treated human peripheral blood monocytes [39]. It may also be possible that reduced IFNγ expression in the early febrile stage of illness resulted in reduced FcγRI expression and hence viral clearance in patients that go on to develop DHF [40]. Studies that address these questions may further clarify the role different types of FcγR play in dengue immunity and pathogenesis.

In conclusion, FcγRI-mediated phagocytosis plays an important role in the removal of antibody-opsonized DENV.
